# Prevalence, Patterns, Prognosis, and Psychosocial Impact of Olfactory and Gustative Dysfunctions Among Saudi COVID-19 Patients

**DOI:** 10.7759/cureus.31743

**Published:** 2022-11-21

**Authors:** Mohammad H Shaheen, Abdullah Alghamdi, Abdullah Alrajhi, Meshal F Khan, Alaa Babkour

**Affiliations:** 1 Otolaryngology, Al-Noor Specialist Hospital, Makkah, SAU; 2 Otolaryngology, Alhada Armed Forces Hospital, Altaif, SAU

**Keywords:** impact, social, psychological, covid-19, ageusia, anosmia

## Abstract

Objective

The objective of this study is to estimate the prevalence of olfactory and gustative dysfunctions (OGD) and analyze their pattern and psychosocial impact among COVID-19 patients.

Method

A cross-sectional study was conducted among 194 confirmed COVID-19 cases at Al-Noor Specialist Hospital between 1 September 2020 and 30 September 2021. A questionnaire was translated and modified from another study to collect the baseline demographic data and medical history; characterization of smell and taste loss separately, including timing, level, practices or treatment used to restore, recovery, and symptom duration; and the psychological impact of OGD using six items to explore the multidimensional impact, such as daily activity, job performance, and social life. A four-point Likert-type agreement scale was used, and an impact score was calculated.

Result

As high as 97.4% and 94.8% of the participants declared having experienced a certain level of olfactory and gustative dysfunction, respectively. In the majority of these cases, the dysfunction occurred after the acute phase of the disease and persisted less than one month after onset. Social life (78.4%), job performance (64.4%), and daily life activities (42.8%) were the most frequently impacted dimensions, and 32% of the participants were deemed to have experienced a high psychosocial impact. Younger participants, females, and certain job categories were significantly more impacted than their counterparts.

Conclusion

OGDs are highly frequent in COVID-19 patients. They are described to be relatively severe and have frequent psychosocial impacts, notably in females and the younger age category. Further research is warranted to determine efficacious preventive and management strategies in order to prevent their long-term impact on wellbeing.

## Introduction

The COVID-19 infection manifests with a wide range of clinical symptoms ranging from asymptomatic cases to severe multiorgan failure [1]. Although the virus has an evident pulmonary tropism, several atypical extrapulmonary symptoms have been identified and added to the list of clinical presentations of the disease [2]. Among these atypical signs, olfactory and gustative dysfunctions (OGDs) were identified as hallmarks of SARS-COV-2 infection since the start of the pandemic and had a high diagnostic value, notably for paucisymptomatic cases [3]. Although described as common since the early series of cases, the prevalence of OGDs varied greatly, ranging between 6% up to 70% [3-8]. These symptoms are characterized by partial (hyposmia and hypogeusia) or total (anosmia and ageusia) loss of the senses of smell and taste, respectively, and were mainly reported in the acute phase of the disease, either isolated, preceding, or associated with other symptoms such as fever, dry cough, or headache [6,9,10].

OGDs are considered among a multiple set of neurological manifestations of COVID-19, owing to the selective tropism of coronaviruses for neurons, using axons as transportation and propagation means. This neuro-invasiveness is explained by three main mechanisms including transneuronal spread, olfactory pathway, and hematogenous spread [11]. Further pathophysiological explanations suggest the role of angiotensin-converting enzyme 2 (ACE2) receptors, which are the entry receptors for the virus into the host cells given their strong binding affinity with the virus spike protein, and which are diffusely expressed in the oral cavity leading to gustative dysfunction [12,13]. Another mechanism involves systematic inflammation leading to deleterious effects on the central nervous system, which explains the multiple neurological symptoms [11]. Finally, olfactory dysfunction may also be explained by damage to the olfactory epithelium, which is commonly caused by the SARS-COV-2 virus as well as other viruses such as rhinovirus and parainfluenza virus [14].

The other characteristic of OGDs is their relatively long persistence after recovery from other symptoms, making them one of the most common long-term complications of COVID-19 [8]. This may result in significant inconvenience for the patients and may impact their quality of life.

In the present study, we estimated the prevalence of OGDs and analyzed their pattern and psychosocial impact. Such data would highlight the clinical significance of these symptoms and provide indications on specific measures to prevent their impact.

## Materials and methods

Design and setting

A cross-sectional study was conducted at Al-Noor Specialist Hospital between 1 September 2020 and 30 September 2021. The study protocol and questionnaire were reviewed and ethically approved by the institutional review board of the Ministry of Health, Makkah region.

The study involved adult individuals who were diagnosed with COVID-19 in any of the outpatient clinics, emergency wards, or hospitalization wards at Alnoor Specialist Hospital between 1 September 2020 through 30 September 2021. Eligibility applied for cases with diagnosis confirmation based on a positive polymerase chain reaction (PCR) test. Patients having no positive PCR test and those aged less than 18 years were excluded.

Sampling

To estimate the sample size, we used the WHO recommendations for the minimum sample size needed for a prevalence study. A previous meta-analysis showed that the prevalence of olfactory dysfunction in COVID-19 patients is 48%. In addition, according to the Ministry of Health (MOH) statistics, around 14,500 patients were confirmed with COVID-19 in Makkah from 1 September 2020 through 30 September 2021. Using a confidence interval of 95%, a standard deviation of 0.5, and a margin error of 5%, the required sample size is 194 participants to be representative of the study population.

Data collection

A semi-structured questionnaire was used to collect the study data, and was adapted from a previous study [15]. The questionnaire was divided into four parts to collect the following data: 1) baseline participant's characteristics including demographic data (age, gender, and occupation) and medical history; 2) characterization of smell loss during COVID-19 infection (six items) including occurrence of anosmia, its level (partial or total), timing (before, concomitant with, or after the other symptoms), practices or treatment used to restore smell, recovery (partial, total, or no recovery), and symptom duration; 3) characterization of taste loss during COVID-19 infection including occurrence of ageusia, type of taste concerned (salty, sweet, etc.), timing (before, concomitant with, or after the other symptoms), practices or treatment used to restore smell, recovery (partial, total, or no recovery), and symptom duration; 4) psychological impact of olfactory and gustative dysfunctions (OGD) using a four-point Likert-type agreement scale (1=disagree, 2=somewhat disagree, 3=somewhat agree, and 4=agree) including six items exploring the multidimensional impact such as daily activity, job performance, and social life. The latter section enabled calculating an OGD impact score, which was computed as the sum of the item scores (range 6-24).

The questionnaire was redacted to the Arabic language and underwent face and content validity by a working group of four local, native Arabic-speaking healthcare professionals, including two consultant physicians and two medical residents. The consented version was pilot tested for clarity and suitability among a group of 10 participants who were not included in the parent study.

Procedure

The questionnaire was edited for an online survey using the SurveyMonkey platform (Momentive, San Mateo, California). The electronic link was sent to participants via WhatsApp.

Statistical methods

Statistical analysis was performed with the Statistical Package for Social Sciences version 21.0 for Windows (IBM Inc., Armonk, New York). Categorical variables are presented as frequency and percentage, while continuous variables are presented as mean ± standard deviation (SD). Factors associated with OGD impact were analyzed by comparing the mean (SD) OGD impact score between the demographic and baseline medical history factors using an independent t-test or one-way analysis of variance (ANOVA) as applicable. A p-value of <0.05 was considered to reject the null hypothesis.

## Results

Baseline participant's characteristics

One hundred and ninety-four confirmed COVID-19 patients were included. The majority were female (70.1%) and in the younger age categories, including <18 years (27.3%), 18-25 years (30.9%), and 25-34 years (15.5%). Students (29.9%), healthcare professions (17.5%), and education (13.9%) were the most frequent occupations. Only 20.6% of the participants declared having a chronic disease. Regarding attitudes toward the COVID-19 vaccine, 16.0% were not expecting to take the vaccine, and 32.5% were hesitating (Table 1).

**Table 1:** Demographic characteristics and medical history (N=194)

Parameter	Category	Frequency	Percentage
Age category (years)	<18	53	27.3
	18-25	60	30.9
	26-34	30	15.5
	35-42	29	14.9
	43-50	15	7.7
	>50	7	3.6
Gender	Female	136	70.1
	Male	58	29.9
Occupation	Student	58	29.9
	The medical section	34	17.5
	Military sector	10	5.2
	Education sector	27	13.9
	Private sector	26	13.4
	Retired	13	6.7
	Free business	13	6.7
	Other	13	6.7
Chronic diseases	No	154	79.4
	Yes	40	20.6
	Nose and sinus allergies	16	8.2
	Asthma and respiratory allergies	16	8.2
	Diabetes	11	5.7
	High blood pressure	12	6.2
	Heart disease	1	0.5
	Chest allergy	1	0.5
	Thyroid dysfunction	2	1
COVID-19 vaccine expectation	Yes, I expect to take the vaccine	56	28.9
	No, I do not expect to take the vaccine	31	16
	Already received the vaccine	44	22.7
	I haven't decided yet	63	32.5

Patterns of smell loss during COVID-19

The prevalence of olfactory dysfunctions during COVID-19 infection was 97.4%, of which 25.8% occurred at the start of the infection, 71.6% after the acute phase, and 82.0% experienced a complete loss of smell. Eighty-six (44.3%) participants declared having attempted to restore their sense of smell using medications such as corticosteroids nasal spray (10.3%), salt water (23.7%), or vitamins (33.5%). On the day of the survey, 36.1% had fully and 54.6% had partially restored their smell, while 6.7% had no recovery. Restoration time was less than three weeks in 55.1%, less than one month in 70.6%, and took longer than one month in 14.4% of the cases (Table 2).

**Table 2:** Patterns of smell loss during COVID-19 (N=19)

Parameter	Category	N	%
Any change in sense of smell during COVID-19 infection?	No loss or weakness of the sense of smell	5	2.6
	Yes, before infection	50	25.8
	Yes, after infection	139	71.6
Was the change or loss of the sense of smell the first sign of the COVID-19 infection?	Before any other symptoms appear	56	28.9
	In conjunction with COVID-19 symptoms	81	41.8
	After other symptoms appeared	57	29.4
Level of smell loss	Full	159	82.0
	Partial	30	15.5
	Not applicable (no smell loss)	5	2.6
Treatments or practices used to restore the sense of smell	None	108	55.7
	Yes	86	44.3
	Cortisone nasal spray	20	10.3
	Saltwater	46	23.7
	Smell recovery exercises	35	18.0
	Vitamins	65	33.5
	Decongestants	11	5.7
Smell restoration	Full	70	36.1
	Partial	106	54.6
	No recovery	13	6.7
	Not applicable (no smell loss)	5	2.6
Duration of smell loss to recovery	1-11 days	46	23.7
	12-21 days	61	31.4
	22-30 days	30	15.5
	>1-3 months	24	12.4
	4-5 months	2	1.0
	>6 months	2	1.0
	I did not recover	13	6.7
	I do not know	11	5.7
	Not applicable (no taste loss)	5	2.6

Patterns of taste loss during COVID-19

The prevalence of gustative dysfunctions during COVID-19 infection was 94.8%, of which 24.7% occurred at the start of the infection, 70.1% after the acute phase, and 58.8% experienced a complete loss of taste. Further characterization of taste loss showed salty (9.3%), sweet (9.3%), and bitter (8.2%) tastes to be the most commonly affected. The majority of participants experienced taste restoration, which was full (22.7%) or partial (63.4%); however, 8.8% had not recovered at the time of the survey. Recovery time was less than three weeks in 55.7% of the cases, less than one month in 70.0%, and longer than one month in 17.5% (Table 3).

**Table 3:** Patterns of taste loss during COVID-19 (N=194)

Parameter	Category	N	%
Any change in the sense of taste during the COVID-19 infection?	No loss or weakness of the sense of smell	10	5.2
	Yes, before infection	48	24.7
	Yes, after infection	136	70.1
Was the change or loss of the sense of smell the first sign of the COVID-19 infection?	Before any other symptoms appear	54	27.8
	In conjunction with COVID-19 symptoms	76	39.2
	After other symptoms appeared	54	27.8
	I haven't lost my sense of taste	10	5.2
Treatments or practices used to restore the sense of smell	None	122	62.9
	Yes	72	37.1
	Cortisone pills	4	2.1
	Taste recovery exercises	8	4.1
	Vitamins	62	32.0
Taste restoration	Full	44	22.7
	Partial	123	63.4
	No recovery	17	8.8
	Not applicable (no taste loss)	10	5.2
Duration of taste loss to recovery	1-11 days	51	26.3
	12-21 days	57	29.4
	22-30 days	20	10.3
	>1-6 months	22	11.3
	>6 months	12	6.2
	I do not know	22	11.3
	Not applicable (no taste loss)	10	5.2

Psychosocial impact of olfactory and gustative dysfunctions

The majority of the participants declared that OGD impacted their social life (78.4%),while 42.8% declared it had significantly affected their daily life (Table 4). The impact score was calculated, and the mean±SD score was 15.54±4.40 (range 6-24). The score was divided into three levels: weak (40, 20.6%), moderate (92, 47.4%), and high impact (62, 32.0%) (Table 4).

**Table 4:** Psychosocial impact of olfactory and gustative dysfunctions OGD: olfactory and gustative dysfunction

Impact dimension	Agree		Somewhat agree		Somewhat disagree		Disagree		Agreement rate
	N	%	N	%	N	%	N	%	
OGD had an effect on my daily activity	43	22.16	40	20.62	58	29.9	53	27.32	42.8%
My job performance is affected by OGD	86	44.33	39	20.1	44	22.68	25	12.89	64.4%
I feel depressed or anxious due to OGD	23	11.86	33	17.01	70	36.08	68	35.05	28.9%
My social life was affected due to OGD	104	53.61	48	24.74	25	12.89	17	8.76	78.4%
OGD impacted my appetite	26	13.4	28	14.43	76	39.18	64	32.99	27.8%
I've been afraid of losing my smell or taste all my life	21	10.82	19	9.79	45	23.2	109	56.19	20.6%

Factors associated with the impact of olfactory and gustative dysfunctions

The OGD impact score was significantly higher among the younger age group (p<0.005), females (p<0.001), and certain occupations, including students, free business, and retired individuals (p<0.001). No significant association of OGD impact was observed with past medical history (p>0.05) (Table 5).

**Table 5:** Factors associated with the impact of olfactory and gustative dysfunctions

Factor	Level	N	Mean	SD	Statistics (test)	p-value
Age (Years)	<18	53	17.21	4.72		
	18-25	60	15.35	4		
	26-34	30	13.73	3.32		
	35-42	29	15.86	4.84		
	43-50	15	13.47	4.34		
	>50	7	15.43	3.65	3.467 (t)	0.005*
Gender	Female	136	16.23	4.5		
	Male	58	13.93	3.72	3.422 (f)	0.001*
Occupation	Student	58	16.93	4.46		
	The medical section	34	13.88	3.98		
	Military sector	10	15.9	2.85		
	Education sector	27	14.93	4.39		
	Private sector	26	14.96	3.56		
	Retired	13	17.46	4.12		
	Free business	13	17.46	4.67		
	Other	13	12	4.44	3.966 (f)	<0.001*
Nose and sinus allergy	No	178	15.71	4.42		
	Yes	16	13.69	3.75	1.770 (t)	0.078
Asthma and respiratory allergy	No	178	15.65	4.47		
	Yes	16	14.31	3.4	1.168 (t)	0.244
Diabetes	No	183	15.44	4.36		
	Yes	11	17.18	4.96	1.138 (t)	0.28
Hypertension	No	182	15.54	4.35		
	Yes	12	15.5	5.25	0.028 (t)	0.978

## Discussion

The present study addressed the clinical significance of OGD in COVID-19 by estimating its prevalence, levels, patterns, and by assessing its perceived psychosocial impact. As high as 97.4% and 94.8% of the participants declared having experienced a certain level of olfactory and gustative dysfunction, respectively. In a majority of these cases, the dysfunction occurred after the acute phase of the disease and persisted less than one month after onset. Social life, job performance, and daily life activities were the most frequently impacted dimensions, and 32% of the participants were deemed to have experienced a high psychosocial impact. Younger participants, females, and certain job categories were significantly more impacted than their counterparts.

There is great variability in the figures of OGD across the studies, reaching up to 74% [3-8,16]. Despite this discrepancy, both anosmia and ageusia were consistently defined as typical clinical features for COVID-19 infection and attracted much attention. Early data from Wuhan reported hypogeusia and hyposmia as the most frequent peripheral neurological symptoms of COVID-19; however, the reported prevalence was less than 6% [17]. An interesting study by Nocini et al. showed a substantial increase in the weekly Google trends scores for the search terms "ageusia" and "anosmia" between the pre-COVID to the COVID-19 period [18]. Nevertheless, findings from the present study are relatively high compared with the literature, concerning almost 100% of the participants. This high prevalence may be due to the recentness of our study influencing the awareness of people about OGD symptoms and consequently increasing their related subjective perception. Other explanations for the variability in the prevalence involve differences in the neurological tropism between the different SARS-COV-2 strains, notably differences in the structure of the spike protein, which result in variable cellular and molecular interplay in the olfactory and gustative epithelia [19].

On the other hand, anosmia and dysgeusia are often associated. A study that estimated the prevalence of anosmia among COVID-19 patients found that dysgeusia was present among 85% of patients with anosmia, and the prevalence of anosmia was 47% [20]. In another cohort, both hyposmia and dysgeusia developed in 33% of the COVID-19 patients, and the two symptoms were concomitantly reported in 27% of the cases (representing 82% of agreement) [21]. This suggests that anosmia and dysgeusia share common pathophysiological mechanisms and or the presence of individual susceptibility to both symptoms.

The duration of both anosmia and dysgeusia was less than three weeks in a majority of the cases in the present study. This is consistent with a study showing a mean anosmia duration of nine days, for a confidence interval 1-21 days. The same study showed that anosmia occurred more than four days, on average, after the disease onset and thus was never the first or second symptom [20]. In another multicenter European study, anosmia developed before the other symptoms in only 12% of the cases [22]. This is consistent with our data showing that approximately 72% of the patient developed anosmia after the acute phase of the disease. However, the timing of OGD onset, by reference to other symptoms, varies greatly in the literature [22,23]. This variability may be explained by the detectability of the symptom, which depends on its severity and the patient's perception, besides eventual pathophysiological mechanisms. On the other hand, the proportion of patients who experience long-term persistence of OGD should not be disregarded. A study showed that as high as 28% and 35% of the cases continued to experience OGD after recovery from COVID-19 [16].

The majority of the participants in the present study declared having used various medications to treat OGDs. Among the most common methods used include vitamins, saltwater (for anosmia), and smell and taste recovery exercise, besides local or systemic corticosteroids. However, the study has not documented whether these methods were recommended or prescribed by a physician or were taken as self-treatment. This is consistent with observations by Lechien et al., who showed that saline irrigation was one of the most frequent methods used to treat OGD, followed by local and oral corticosteroids [22]. The efficacy of corticosteroids in anosmia and ageusia was not evidenced in clinical trials and is, therefore, not recommended [24]. Regarding smell exercise, it consists of a simple and safe form of sensory training that has shown efficacy in restoring smell after post-traumatic and post-infectious olfactory dysfunction [25,26].

The high prevalence, relative duration, and inconvenience of OGD have raised concerns about their impact on individuals' quality of life and psychosocial wellbeing. The present study showed that OGD was frequently associated with a perceived impact on social life, job performance, and daily life activity. There is overwhelming evidence about the COVID-19 impact on psychological and social wellbeing, showing a range of disorders such as anxiety, depression, and post-traumatic stress syndrome, along with social and financial hardships. Such an impact was multifactorial and concerned all age and social and professional categories of the population [27-29]. Nevertheless, not many studies investigated the specific psychosocial impact of OGD during the pandemic. Interestingly, a study involving 15,821 COVID-19 patients demonstrated a significant association between sensory dysfunctions, including anosmia and ageusia, with an increased risk of developing depression and suicidal ideation, which may persist in the long term [30]. Although this may concord with our findings, establishing the causal relationship of OGD with psychological and social impact requires further evidence. It remains, however, necessary to consider these symptoms, particularly in the case of long-term persistence, as these may alter the quality of life.

Limitations

The present study is limited by the cross-sectional design and the self-reported methodology, which entails a high risk of information bias, notably in determining the chronology and appraising the authenticity and severity of the symptoms.

## Conclusions

Olfactory and gustative dysfunctions are highly frequent in the present cohort of COVID-19 patients, appear more frequently after the other symptoms, and persist for less than one month in a majority of the cases. They are described to be relatively severe and have frequent psychosocial impacts, notably in females and the younger age category. Further research and clinical trials are warranted to explore their pathophysiology and best treatment options, along with preventing their long-term persistence and impact on individuals' wellbeing.
